# Who wants to be a psychiatrist? Northern Ireland foundation doctors (2006 - 2018) positive towards psychiatry as career choice

**DOI:** 10.1192/bjo.2021.659

**Published:** 2021-06-18

**Authors:** Michael Doris, Amy Grimason, Damien Hughes, Lorraine Parks, Angela Carragher

**Affiliations:** 1 Belfast HSC Trust; 2 South Eastern Health and Social Care Trust; 3 Belfast HSC Trust; 4 Southern Trust

## Abstract

**Aims:**

Recruitment into psychiatry remains a major issue nationally despite recent progress with the #choose psychiatry scheme. Here we look to establish why Northern Ireland (NI) has been able to have 100% fill rates by speaking to the people who have work in the frontline of psychiatry. What is done differently in NI and are there lessons that could benefit other regions?

**Background:**

NI presents itself as an anomaly – In a region that only attracts 31.8% of F2s to enter into any training programme, Core psychiatry has been consistently oversubscribed for many years. One difference is the allocation of trainees in the Foundation programme. NI offers psychiatry placements to 33% of F2 doctors with none in the F1 year.

**Method:**

All doctors of any grade working in psychiatry who had been through the Foundation programme since 2006 were asked to complete a survey on their foundation experience and reasons for choosing a career in psychiatry using SurveyMonkey technology. Qualiative and quantiative data was collected and analysed.

**Result:**

in total 67 doctors from CT1 to Consultant and SAS doctors responded, including over 60% of all current trainees, providing a huge amount of information. Remarkably, 45% of psychiatry doctors working in NI surveyed hadn't considered a career in psychiatry until their foundation placement. NI is the only region in the UK that does not have an F1 placement in Psychiatry. Over 80% of doctors here feel that this is a positive. White space answers revealed other aspects of training that positively influenced them to choose psychiatry including a reputation for high quality training, as well as close links between the local medical school, the local brach of the Royal College of Psychiatrists and the NI deanery.

**Conclusion:**

This study is the first to examine the reasons behind psychiatry's success in NI. The quality of the training scheme locally and presence of an excellent training to service provision balance were also mentioned. This study supports the presence of psychiatry in the F2 year only.

